# Biofilm formation capacity and presence of virulence factors among commensal *Enterococcus* spp. from wild birds

**DOI:** 10.1038/s41598-019-47602-w

**Published:** 2019-08-01

**Authors:** Dagmara Stępień-Pyśniak, Tomasz Hauschild, Urszula Kosikowska, Marta Dec, Renata Urban-Chmiel

**Affiliations:** 1 Department of Veterinary Prevention and Avian Diseases, Institute of Biological Bases of Animal Diseases, Faculty of Veterinary Medicine, University of Life Sciences in Lublin, Lublin, Poland; 2 Department of Microbiology, Institute of Biology, University of Bialystok, Białystok, Poland; 3 Department of Pharmaceutical Microbiology with Laboratory for Microbiological Diagnostics, Medical University in Lublin, Lublin, Poland

**Keywords:** Bacterial pathogenesis, Biofilms

## Abstract

Enterococci are opportunistic pathogens that can form biofilms during infections and many virulence determinants are involved in this process. Although the virulence factors are often analysed in *Enterococcus* spp. from humans and food animals, little is known about gut enterococcal isolates from wild birds. Therefore, the determination of virulence factors among enterococci isolated from wild birds may provide new information about a possible source of infection for humans and animals or vice versa via the environment. We analysed different phenotypic and genotypic traits in enterococci from wild birds related to potential virulence in humans and animals and to evaluate biofilm formation and its relationship to virulence genes. The *E. faecalis* isolates were characterised by greater frequency of biofilm formation in BHI than *E. faecium*. There was a correlation between hydrophobicity and biofilm formation in BHI broth in *E. faecalis*. None of the isolates was haemolytic. The presence of some adhesion and gelatinase genes was detected in biofilm-positive isolates. The enterococcal pathogenic factors (*esp*, *hyl*, and *cyl* operon genes) did not seem to be necessary or sufficient for production of biofilm by analysed bacteria. *Enterococcus* species isolated from wild birds should be considered as a possible source of some virulence determinants.

## Introduction

Biofilm is considered as an essential factor in the pathogenesis of various opportunistic bacteria, e.g. during enterococcal infections^1^. Moreover, many virulence determinants in *Enterococcus* spp. are involved in the pathological process and the ability to growth in biofilm^2^.

An important step in induction of infection and/or biofilm production is the adhesion of bacterial cells to host tissues. Surface proteins that bind to receptors on the surface of eukaryotic cells or extracellular matrix proteins are adhesion factors; in enterococcal species these factors mainly include enterococcal surface protein (encoded by *esp*), aggregation substance (encoded by *agg* or *asa1*), and collagen-binding protein (encoded by *ace*)^1^. Production of adhesin-like *E. faecalis* and *E. faecium* endocarditis antigen A (encoded by *efaA*_*fs*_ and *efaA*_*fm*_, respectively) is also involved in enterococcal adhesion to biotic and abiotic surfaces. Moreover, expression of pili (encoded by *ebpABC* locus, *srt*, and *pil*) on the cell surface is considered as an important virulence factor facilitating cell adhesion and biofilm formation^2^.

Enterococcal cells are capable of communicating via peptide pheromones (e.g. encoded by *cpd*, *cob*, and *ccf*), which are secreted by recipient cells to induce the conjugative apparatus of donor cells. In this way they mediate the transfer of pheromone-responsive plasmids, which may carry virulence genes that promote biofilm formation or regulation. In response to increased cell population densities, important virulence factors in *Enterococcus* spp. are regulated by quorum sensing, including the *fsr* (faecal streptococci regulator) locus. Changes in the activity of particular genes lead to maturation of the biofilm and the appearance of appropriate phenotypic traits, depending on the conditions. In the case of *Enterococcus* spp., cytolysin, i.e. a haemolytic exotoxin (encoded by *cyl*), gelatinase (encoded by *gelE*), serine protease (encoded by *sprE*), and hyaluronidase (encoded by *hyl*) are the most frequent virulence factors affecting host cells by their toxic or destructive effects^3^.

Enterococci are Gram-positive elements of the gut microbiota and significant agents of opportunistic infections in animals and humans^4,5^. *Enterococcus* spp. from avian species associated with diseases include mainly *Enterococcus faecalis*, *E. faecium*, *E. durans*, *E. hirae* and *E. cecorum*^6–8^. The collection of *Enterococcus* isolates from wild birds analysed in this study, especially *E. faecalis* and *E. faecium*, are resistant to multiple antibiotics and represent different epidemic clones (e.g. CC17, CC81, CC116)^9,10^. The same clones have previously been found in hospitalised patients in Poland and other European countries, as well as globally in unhospitalised patients, livestock, pets, food and wastewater^11–13^. For bacteria to be pathogenic, in addition to antibiotic resistance they must also possess virulence factors^14^. In addition, enterococci are naturally able to acquire, accumulate, and transmit extrachromosomal elements encoding virulence traits^15^. Thus, wild birds may be considered a source of enterococci that are potentially pathogenic as well as resistant to antibiotics. Transmission of such enterococcal strains from wild birds to humans and animals is possible via environment, such as: water polluted with faeces, soil, dietary patterns in wild animals/birds, meat or direct contact during hunting in humans^1^. Taking into account migration of birds, the same etiological agent can occur in distant places.

Monitoring faecal *Enterococcus* spp. as indicator bacteria in different populations, including wild birds makes it feasible to compare the prevalence of virulence in determined STs/CC and to detect the transfer of potentially pathogenic strains from animals to humans and vice versa. However, a few reports of the prevalence of virulence factors in antibiotic resistant enterococci from wild animals have been published^16–19^.

The objective of this work is to analyse different phenotypic and genotypic traits in enterococcal isolates from the gut microbiota of different wild birds related to potential virulence in humans and animals and to evaluate *in vitro* biofilm formation and its relationship to presence of virulence genes.

## Results

### Haemolytic and gelatinase activities

None of the isolates was haemolytic on 5% horse blood agar. Gelatinase was produced by the 27 isolates of *E. faecalis* (100%), one isolate of *E. faecium* (20%), and one isolate of *E. hirae* (33.3%) of the *gelE*-positive strains (Table S1).

### Hydrophobicity and biofilm formation

The *in vitro* hydrophobicity test revealed twenty strains with hydrophobicity higher than 50%; eighteen of them represented *E. faecalis* and the other two strains belonged to the species *E. faecium* and *E. casseliflavus*. The *E. faecalis* strains showed significantly higher hydrophobicity than *E. faecium* (*P* < 0.001; Chi-square test), i.e. as many as 33.3% of the *E. faecalis* isolates displayed hydrophobicity at the level of 100% and another 33.3% exhibited %H in the range of 50–70%. Detailed data are shown in Table S1.

Biofilm formation by the tested *E. faecalis* and *E. faecium* detected with the microtitre plate method in BHI broth supplemented with 2% glucose was statistically significantly higher than in TSB broth supplemented with 1% glucose (*P* < 0.001; Chi-square test). Both species differed significantly in biofilm formation in TSB broth supplemented with 1% glucose (*P* = 0.00886; Chi-square test). However, the *E. faecalis* and *E. faecium* species did not differ in biofilm formation in BHI broth supplemented with 2% glucose (*P* = 0.59083; Fisher’s exact test) (Table S1). Among a total of isolates the ability to biofilm formation in BHI was observed in 87% (47 isolates); 70.4% (38 isolates) were classified as moderately or strongly adherent and 16.7% (9 isolates) were weakly adherent. Seven isolates (13%) showed no ability to form biofilm in BHI broth (Table 1).

**Table 1:** Association between biofilm-forming strength in BHI Broth supplemented with 2% glucose and TSB Broth with 1% glucose and *Enterococcus* species (no. of strains/%).

Biofilm strength	*E. faecalis* (*n* = 27)		*E. faecium* (*n* = 18)		*E. hirae* (*n* = 5)		*E. durans* (*n* = 2)		*E. casseliflavus* (*n* = 2)	
	BHI^a^	TSB^b^	BHI^a^	TSB^b^	BHI^a^	TSB^b^	BHI^a^	TSB^b^	BHI^a^	TSB^b^
No biofilm	4/14.8	9/33.3	3/16.7	14/77.8	—	2/40	—	2/100	—	2/100
Weak	3/11.1	9/33.3	3/16.7	3/16.7	2/40	3/60	1/50	—	—	—
Moderate	13/48.1	6/22.2	8/44.4	—	2/40	—	1/50	—	1/50	—
Strong	7/25.9	3/11.1	4/22.2	1/5.6	1/20	—	—	—	1/50	—
Total biofilm producers	23/85.2	18/66.7	15/83.3	4/22.2	5/100	3/60	2/100	0	2/100	0

^a^BHI broth supplemented with 2% glucose; ^b^TSB broth supplemented with 1% glucose.

In the *E. faecalis* strains, there was a link between biofilm formation in BHI broth supplemented with 2% glucose and hydrophobicity (*P* = 0.00718; Fisher’s exact test). However, there was no correlation between biofilm formation and hydrophobicity in the *E. faecium* strains in the same conditions (*P* = 0.83333; Fisher’s exact test).

### Detection of virulence genes

The prevalence of virulence genes detected in all isolates is shown in Table 2. No *esp*, *hyl*, *cylA*, *cylB*, *cylM*, and *cyl*_*L*_ genes were detected in any of the tested isolates.

**Table 2:** Incidence of virulence factors in enterococci isolated from cloacal swabs of wild birds.

Virulence factor	*E. faecalis* (*n* = 27) No. (%)	*E. faecium* (*n* = 18) No. (%)	*E. hirae* (*n* = 5) No. (%)	*E. durans* (*n* = 2) No. (%)	*E. casseliflavus* (*n* = 2) No. (%)
*ebpA*	19 (70.4)	4 (22.2)	1 (20)	0 (0)	0 (0)
*ebpB*	23 (85.2)	0 (0)	0 (0)	0 (0)	0 (0)
*ebpC*	20 (74.1)	5 (27.8)	3 (60)	0 (0)	2 (100)
*pil*	7 (25.9)	0 (0)	1 (20)	0 (0)	0 (0)
*srt*	26 (96.3)	6 (33.3)	3 (60)	0 (0)	0 (0)
*ace*	26 (96.3)	5 (27.8)	1 (20)	0 (0)	0 (0)
*agg*	9 (33.3)	1 (5.6)	0 (0)	0 (0)	0 (0)
*asa1*	9 (33.3)	2 (11.1)	1 (20)	0 (0)	0 (0)
*esp*	0 (0)	0 (0)	0 (0)	0 (0)	0 (0)
*efaAfs*	27 (100)	0 (0)	0 (0)	0 (0)	0 (0)
*efaAfm*	0 (0)	18 (100)	0 (0)	0 (0)	0 (0)
*gelE*	27 (100)	5 (27.8)	3 (60)	0 (0)	0 (0)
*sprE*	27 (100)	5 (27.8)	3 (60)	0 (0)	0 (0)
*hyl*	0 (0)	0 (0)	0 (0)	0 (0)	0 (0)
*cylA*	0 (0)	0 (0)	0 (0)	0 (0)	0 (0)
*cylB*	0 (0)	0 (0)	0 (0)	0 (0)	0 (0)
*cylM*	0 (0)	0 (0)	0 (0)	0 (0)	0 (0)
*cylL*_*L*_	0 (0)	0 (0)	0 (0)	0 (0)	0 (0)
*fsrA*	27 (100)	5 (27.8)	3 (60)	0 (0)	0 (0)
*fsrB*	27 (100)	5 (27.8)	1 (20)	0 (0)	0 (0)
fsrC	27 (100)	5 (27.8)	3 (60)	0 (0)	0 (0)
*cpd*	27 (100)	5 (27.8)	3 (60)	0 (0)	0 (0)
*cob*	27 (100)	2 (27.8)	2 (40)	0 (0)	0 (0)
*ccf*	27 (100)	17 (27.8)	4 (80)	2 (100)	2 (100)

The screening results of pili components are summarised as follows: *ebpA* was present in *E. faecalis* (70.4%, 19 isolates), *E. faecium* (22.2%, 4 isolates) and *E. hirae* (20%, 1 isolate); *ebpB* was found only in *E. faecalis* (85.2%, 23 isolates); *ebpC* was present in *E. faecalis* (74.1%, 20 isolates), *E. faecium* (27.8%, 5 isolates), *E. hirae* (60%, 3 isolates), and *E. casseliflavus* (100%, 2 isolates); *pil* was detected in *E. faecalis* (70.4%, 19 isolates) and *E. hirae* (20%, 1 isolate), and *srt* was present in *E. faecalis* (96.3%, 26 isolates), *E. faecium* (33.3%, 6 isolates), and *E. hirae* (60%, 3 isolates). There was a correlation between biofilm formation in BHI broth supplemented with 2% glucose and the presence of the *pil* gene in the enterococcal bacteria (*P* = 0.03010; Chi-square test).

*E. faecalis* and *E. faecium* isolates differ in the number of *ebpA*, *ebpB*, *ebpC* and *srt* genes. The prevalence of *ebpA* (*P* = 0.00422; Chi-square test), *ebpB* (*P* = 0.0000; Chi-square test), *ebpC* (*P* = 0.00586; Chi-square test) and srt (*P* = 0.0000; Chi-square test) genes were significantly higher among *E. faecalis* isolates (19strains/70.4%, 23 strains/85.2%, 20 strains/74.1% and 26 strains/96.3%, respectively) than among *E. faecium* isolates (4 strains/22.2%, 0 strains/0%, 5 strains/27.8% and 6 strains/33.3%, respectively). The presence of *pil* gene between *E. faecalis* and *E. faecium* isolates was not found to be statistically significant (*P* = 0.0535; Fisher’s exact test). In addition, the pili genes were also found in *E. hirae* and *E. casseliflavus* isolates. The predominant gene was *ebpC (E. hirae* – 3 isolates/60% and *E. casseliflavus* – 2 isolates/100%). Moreover, the presence of *ebpA, pil* and *srt was seen in only one E. faecalis isolate*. Nine (33.3%) of *E. faecalis* and one (5.6%) *E. faecium* isolates co-harboured the genes *agg* and *asa1*. Additionally, the *asa1* gene was found in one (5.6%) *E. faecium* and one (20%) *E. hirae* isolate. The *E. faecalis* and *E. faecium* isolates did not differ statistically for the presence of the *asa1* gene (*P* = 0.08689; Fisher’s exact test); however, there was a difference in the presence of the *agg* gene (*P* = 0.02909; Fisher’s exact test).

The *efaA*_fs_ and *efaA*_fm_ genes were found in all *E. faecalis* and *E. faecium* isolates, respectively. None of these genes was detected in *E. hirae*, *E. casseliflavus*, and *E. durans*.

The presence of the *gelE* and *sprE* genes associated with the *fsrABC* locus was detected in all 27 *E. faecalis* isolates, 5 *E. faecium*, and 1 *E. hirae*. Additionally, *fsrA* and *fsrC* were found in two *gelE*- and *sprE*-positive *E. hirae* isolates. Moreover, the *ace* gene was detected in the *fsrABC*-positive isolates, except one *E. faecalis*.

The sex pheromone determinants (*cpd*, *cob*, *ccf*) were widespread in the analysed *E*. *faecalis* isolates (100% strains). The *ccf* gene was also detected in all but one *E. faecium*. Moreover, the *cpd* and *cob* determinants were showed in *E. faecium* (five and two isolates, respectively) and *E. hirae* (tree and two isolates, respectively).

The genotypic patterns of the virulence factors detected in the isolates are shown in Table S1.

## Discussion

The study was conducted to determine the prevalence of biofilm-forming ability among gut enterococci isolated from wild birds and its correlation with virulence genes.

There are no literature reports of biofilm formation and hydrophobicity in enterococci isolated from wild birds. In *in vitro* conditions, the microplate method was found to be the most common and effective approach for detection of biofilm production^20^. As was noted during our studies, the composition of the medium was important to show the ability of enterococci to *in vitro* biofilm formation. Other authors also pointed to similar dependencies^20,21^.

Moreover, we observed that the *E. faecalis* strains produced biofilm in BHI more often than *E. faecium*. Leuck *et al*.^22^ recently tested the biofilm-forming ability of *E. faecalis* isolates on polystyrene dishes. The authors reported that many of the clinical isolates showed low-level biofilm formation even when different types of media were used. However, they observed that clinical isolates were able to form biofilms on a relevant tissue surface.

Additionally, we found a correlation between hydrophobicity and biofilm formation in only *E. faecalis*. In addition, the *E. faecalis* isolates showed significantly higher hydrophobicity than *E. faecium*. It is known, that bacterial cell surface hydrophobicity is important for the interactions between the bacterium and host epithelial cells^23^. The hydrophobicity of the enterococcal cell surface is increased by the presence of aggregation substances. Strains possessing the *agg* or *asa1* gene may form large cell aggregates during infection^24^. In the present study, the genetic determinants of aggregation substances were most frequently detected in *E. faecalis*, followed by *E. faecium* and *E. hirae*. The aggregation substance is a sex pheromone plasmid-encoded surface protein. Some of *E. faecalis* strains with all sex pheromone genes (*cpd, cob*, and *ccf*) exhibited the presence of the *agg* and *asa1* genes as well. Similarly, Martin and coworkers^25^ noted the presence of the *agg*, *cpd*, and *ccf* genes in all *E. faecalis* isolates. Additionally, production of sex pheromones by *E. faecalis* may favour acquisition of antibiotic resistance and virulence from other enterococci, resulting in increased virulence. In the present study, a lower proportion of sex pheromone genes were observed in *E. faecium* in comparison with *E. faecalis* isolates. However, the sex pheromone genes in *E. faecium* may reflect sequence divergence, which may explain this result. Importantly, Eaton and Gasson^26^ showed that virulence determinants can be transferred from pathogenic strains to non-pathogenic strains (used as starters in food). The authors did not achieve transfer into *E. faecium* strains, although sex pheromone cross talk between *E. faecium* and *E. faecalis* has been demonstrated^27^.

Our finding corroborates the results reported by Martin and coworkers^25^, who demonstrated that all *E. faecalis* and *E. faecium* strains from Wood Pigeon (*Columba palumbus*) carried the *efaA*_*fs*_ and *efaA*_*fm*_ virulence genes, respectively, while the *ace* gene was more often found in *E. faecalis* strains from wild birds in Poland than from Partridges in Portugal^16^. Both *efaA*_*fs*_ and *ace* genes play a role in the pathogenesis of endocarditis, whereas the role of *efaA*_*fm*_ is yet unknown.

Some authors demonstrated that the presence of pili genes in enterococcal strains is required for the establishment of the first step of infection^28^. As shown in our study, most of the analysed *Enterococcus* spp. carried these genes; however, a correlation was only observed between biofilm formation in BHI with 2% glucose medium and the presence of the *pil* gene.

No *esp*, *hyl*, *cylA*, *cylB*, *cylM*, *cyl*_*L*_ genes were detected in any of the tested strains in our studies. The absence of the *cyl* operon was associated with negative *β*-haemolysis. Similarly, no *β*-haemolytic activity was found in enterococal strains from wild boars (*Sus scrofa*) in Spain, although the strains contained different combinations of *cyl* genes, in contrast to our results^17^. Olsen and coworkers^29^ showed that the *cylA* gene was detected more often in clinical than in commensal poultry isolates, where none of the isolates was haemolytic. As indicated previously, cytolysin activity requires the presence of the whole *cyl* operon (*cyl L*_*L*_*L*_*S*_*ABM*)^26,30^. This was also confirmed in the study conducted by Silva and coworkers^31^. Cytolysin exerts activity against a broad spectrum of cell types including a wide range of Gram-positive bacteria, eukaryotic cells such as human, bovine, and horse erythrocytes, retinal cells, polymorphonuclear leukocytes, and human intestinal epithelial cells^32^.

As in our results, Silva and coworkers^16^ did not detect any *esp* genes in enterococci isolated from Partridge (*Alectoris rufa*), whereas this gene was described seven years later in six *vanA*-positive *E. faecium* isolated from the same bird species^33^. Additionally, the *esp* gene was found in two *vanA/B2*-positive *E. faecalis* strains^34^. Interestingly, the *esp* gene in *E. faecalis* is located on a large genetic component (150 kb) characterised by all features of the pathogenicity island, whose presence is characteristic for multiresistant isolates, including vancomycin-resistant^35^. Indeed, the *esp* gene is present predominantly in strains associated with infections and hospital outbreaks^36,37^.

In contrast to the isolates from the wild birds analysed in our study, the *hyl* gene was detected in two and one *E. faecium* strains isolated from Wild Boars and Partridges, respectively^16,17^. Moreover, the *hyl* gene was found in five *van*A-positive *E. faecium* strains from wild partridges^33^ and three *vanA/vanB2 E. faecalis* strains from two Cattle Egrets and one Common Ringed Plover^34^. Gram-positive genera capable of elaborating hyaluronidase are able to cause infections initiated at a mucosal or skin surface of either humans or animals^38^.

It was reported in our study that all *E. faecalis* strains that exhibited gelatinase activity harboured the *gelE*, *sprE*, and *fsrABC* genes, which is in agreement with results reported by other authors^17,18^. Additionally, only one *E. faecium* and one *E. hirae* strains had gelatinase activity and harboured *gelE*, *sprE*, and all *fsr* operon genes. A discrepancy between the presence of *gelE*^19^ including *sprE* and *fsrABC* genes^18^ and production of gelatinase in enterococci was also observed which coincide with our results. Gelatinase (*gelE*) is co-transcribed with serine protease (*sprE*) and regulated by the quorum-sensing *fsr* locus. It can also cleave sex pheromones, which are known to be potent chemo-attractants^39^ and might therefore modulate the host response.

Many authors indicate the presence of numerous genes and virulence factors in both pathogenic and opportunistic bacteria^28,40–42^. However, they do not specify which of them may have of greatest importance for pathomechanism of infections, because it is a complex and multi-stage process and depends of many factors, including bacterial virulence as well as the conditions of the host and habitat and the presence of another components of microbiota. Similarly, it is difficult to do so in the case of enterococci isolated during our studies from the gut microbiota of wild birds. However, based on our findings, commensal enterococci from the wild birds had some virulence determinants and could be a source of potential pathogenic strains for humans and animals, especially that some of them were determined as antibiotic resistant epidemic clones. This hypothesis can be confirmed by the recent results of investigations of virulence factors in vancomycin-resistant enterococci from wild birds obtained by Ben Yahia and coworkers^34^, showing that *van*A/*van*B2 *E. faecalis* strains can also harbour the important virulence determinants.

## Conclusion

In conclusion, the data presented in this study can help to elucidate the prevalence of virulence factors in enterococcal isolates from wild birds in Poland and indicate that *Enterococcus* species should be considered as a possible source for virulence determinants. None of the analysed genes should be considered definitive markers of pathogenicity in the tested bacteria. Moreover, the results of this study showed that the presence of pathogenic factors such as the *esp*, *hyl*, and *cyl* operon genes did not seem to be necessary or sufficient for the production of biofilm by enterococci in the analysed conditions. However, the presence of some adhesion and gelatinase genes has been detected in biofilm-positive isolates. It appears that many environmental conditions, e.g. the medium composition, and genetic factors may be associated with the pathomechanism and production of biofilm by enterococci. Therefore, the environment, e.g. organs outside the gastrointestinal tract where the bacteria live, affects their surface activity and intercellular interactions. In some cases, our strains also possessed silent virulence genes.

## Methods

### Collection of strains

The collection of 54 *Enterococcus* isolates (*E. faecalis*, 27 isolates; *E. faecium*, 18 isolates; *E. hirae*, 5 isolates; *E. durans*, 2 isolates; and *E. casseliflavus*, 2 isolates) from cloacal swabs of 52 free-living birds representing 25 species was studied (Table S1). The swabs for bacteriological analysis were collected from birds after their delivery to the Centre for Rehabilitation of Wild Birds, University of Life Sciences in Lublin, which receives injured or weak birds. The cloacal samples from the birds were collected by a veterinarian as part of his work and based on the authorization to collect biological material for research purposes by the Regional Directorate for Environmental Protection (WPN.6401.45.2015.MPR.1). The study was conducted in an ethical and responsible manner, in full compliance with all relevant codes of experimentation and legislation. Enterococci were isolated on Bile Esculin Azide Lab-Agar (Biocorp, Warsaw, Poland) at 37 °C for 24**–**48 h and identified to the species level by Ultraflextreme Matrix Assisted Laser Desorption Ionization Time of Flight Mass Spectrometry (MALDI-TOF MS) with MALDI-Biotyper 3.0 software (Bruker Daltonics, Bremen, Germany) and *rpoA* gene sequencing, as previously described^43^. In addition, antibiotic resistance and genetic diversity of analysedstrains were determined previously^9,10^. The isolates were stored at −80 °C in Brain Heart Infusion Broth (Oxoid, Basingstoke, Hampshire, UK) with 20% sterile glycerol for further analysis.

### Haemolysin and gelatinase activity screening

Haemolysis was evaluated by plating the strains on Columbia Agar Base (OXOID, Basingstoke, Hampshire, UK) supplemented with 5% defibrinated horse blood (Pro Animali Company, Wroclaw, Poland). The plates were incubated at 37 °C for 24 h in aerobic conditions. A positive result was indicated by the formation of haemolytic (clear) zones around the colonies. *E. faecalis* ATCC29212 (LGC Standards, Łomianki, Poland) was used as a positive control.

Gelatinase production was detected by inoculating the *Enterococcus* strains onto Trypticase Soy Agar (OXOID, Basingstoke, Hampshire, United Kingdom) containing 3% gelatine (Avantor Performance Materials, Gliwice, Poland). The appearance of a clear halo around the colonies after incubation at 37 °C for 24 h in aerobic conditions followed by refrigeration at 4 °C for 30 min was considered to be a positive indication of gelatinase production. *E. faecalis* ATCC29212 (LGC Standards, Łomianki, Poland) was used as a positive control.

### Hydrophobicity and biofilm assays

Cell surface hydrophobicity was determined using the method developed by Dec and coworkers^44^. Each isolate was subcultured on Columbia Agar Base (OXOID, Hampshire, UK) supplemented with 5% defibrinated horse blood (Pro Animali Company, Wroclaw, Poland) at 37 °C. 24-h cultures were harvested and suspended in 5 ml of 0.9% NaCl to an optical density (OD_600_) of 0.8–1.0 (A_0_). Then, xylene (1.7 ml) was added to glass test tubes and the mixtures were vortexed vigorously for 90 s. After phase separation (ca. 15 min.), the optical density of the aqueous phase (A) was measured again and compared with the initial value. The percentage of cell surface hydrophobicity (%H) of the strain adhering to xylene was calculated using the equation: %H = [(A_0_ − A)/A_0_] × 100. Strains with hydrophobicity equal or higher than 50% were considered hydrophobic.

Biofilm assays were conducted based on a method described by Stepanović and coworkers^20^ using Tryptic Soy Broth (TSB) (Oxoid, Hampshire, UK) supplemented with 1% glucose and Brain Heart Infusion (BHI) (Oxoid, Hampshire, UK) supplemented with 2% glucose. Each isolate was subcultured on Columbia Agar Base (OXOID, Hampshire, UK) supplemented with 5% defibrinated horse blood (Pro Animali Company, Wroclaw, Poland) at 37 °C. After verification of the purity of the strain, a few colonies with identical morphology are suspended in physiological saline. Then, the turbidity of the bacterial suspension was adjusted to match turbidity comparable to that of the 0.5 McFarland standard (~10^8^ CFU/ml) using a densitometer Biosan DEN-1 (Biogenet, Józefów-Otwock, Poland). Then, for each strain tested, 20 µl of bacterial suspensions were transferred to four wells of two separate sterile flat-bottomed 96-well polystyrene microtitre plates containing 180 µl of TSB supplemented with 1% glucose and 180 µl of BHI supplemented with 2% glucose, respectively. For the negative control, 200 µl of broths (TSB and BHI, both with glucose) were dispensed into eight vertical wells per plate. The plates were incubated under stationary aerobic conditions at 37 °C. After incubation for 24 hours, the broths were carefully removed. The wells were gently washed three times with phosphate-buffered saline (PBS, pH 7.2). Following every washing step, the wells were emptied by flicking the plates. Prior to biofilm staining, the plates were left at room temperature for drying in an inverted position overnight. The adherent biofilm layer formed in each microtitre plate well was stained with 200 µl of 0.1% crystal violet solution in water for 15 min at room temperature. After staining, the stain was aspirated with a pipette and excess stain was rinsed off by placing the microtitre plate under running tap water. Washing was continued until the washings were free of the stain. After the microplates were dried at room temperature, the dye bound to the cells was resolubilised with 200 µl of 96% ethanol per well for 30 min without shaking. The optical density (OD) of the resolubilised crystal violet was then measured at 570 nm (OD_570_) using a microplate reader (Bio-Rad, Model 680). Each assay was performed in quadruplicate on three occasions for 12 readings for each strain. Wells containing uninoculated medium served as negative controls to determine the background optical density. After subtracting the mean background OD_570_ readings, the 12 optical density readings per strain were averaged to obtain the mean OD_570_ reading for each strain. Based on the bacterial biofilm, the isolates were classified into four categories: non-biofilm producers, weak, moderate, or strong biofilm producer. The isolates were classified as follows: OD < ODc = non-biofilm producers (category I); ODc < OD < 2ODc = weak biofilm producers (category II); 2ODc < OD < 4ODc = moderate biofilm producers (category III); and OD > 4ODc = strong biofilm producers (category IV). The cut-off OD (ODc) was defined as three standard deviations above the mean OD of the negative control.

### PCR

DNA was extracted using a commercial GeneMATRIX Bacterial & Yeast Genomic DNA Purification Kit (Eurx, Poland). To improve the nucleic acid extraction efficiency, lysozyme was used in the enzymatic lysis step. The presence of genes encoding putatitve virulence factors of the *Enterococcus* strains was evaluated using PCR with specific primers that encode the endocarditis- and biofilm-associated pili genetic locus (*ebpABC)* and an adjacent downstream sortase-encoding gene (*srt*), pili (*pil*), aggregation substance (*agg* and *asa1*), collagen binding protein (*ace*), enterococcal surface protein (*esp*), enterococcal endocarditis antigen (*efaAfs* for *E. faecalis* and *efaAfm* for *E. faecium*), gelatinase (*gelE*), serine protease (*sprE*), hyaluronidase (*hyl*), cytolysin (*cylA*, *cylB*, *cylM* and *cylL*_*L*_), the quorum sensing locus *fsr* (*fsrA*, *fsrB*, *fsrC*), and sex pheromones (*cpd*, *cob* and *ccf*). These virulence genes were chosen, because they are detected most frequently in clinical isolates of enterococci. All primer sequences are listed in Table S2. PCR was performed on a T100 thermal cycler (Bio-Rad Laboratories Inc., Hercules, CA, USA) in a final volume of 25 µl containing 1 µl (~20 ng) of DNA as template; 2.5 µl of reaction buffer (10x); 0.2 µl of Taq DNA polymerase (5U/1 µl); 1 µl of each of the two primers (10 pmol/1 µl, Genomed, Warsaw, Poland), 0.2 µl of 25 mM dNTPs MIX; and PCR pure water to a final volume. The reagents used in the PCR mixtures were purchased from AmpliKIT Allegro Taq (Novazym, Poznań, Poland).

The initial 5-min denaturation step at 95 °C was followed by 35 cycles of 1-min denaturation at 95 °C, annealing at the different temperatures shown in Table 1 for 1 min, and an extension at 72 °C for 1 min, followed by final extension at 72 °C for 8 min. The PCR products were analysed in 1.5% agarose–Tris–borate–EDTA gel containing 0.5 mg of ethidium bromide per ml and then visualised with a gel imaging analysis system with Quantity One software (Bio-Rad; Hercules, CA, USA). *E. faecalis* ATCC29212 (*ace, asa1, gelE, efaA*_*fs*_*, cpd, cob, ccf, cylA*), *E*. *faecalis* P33 (*cylA*, *cylB*, *cylM, cylL*_*L*_, *esp*), *E. faecalis ATCC27285* (*agg*)*, E. faecalis OG1RF* (*ace, fsrA, fsrB, fsrC, gelE, sprE, ebpA, ebpB, ebpC, pil, srt*)*, E. faecium* ATCC19434 (*efaA*_*fm*_) were used *as* positive controls. *E. faecalis* P33 (sequence type 16) came from our collection and was isolated from one-day old broiler chickens with yolk sac infection. Nuclease-free water was used as a negative control.

### Statistical analysis

All statistical analyses were performed using STATISTICA program version 13.1 (StatSoft Inc., 2014, Tulsa, OK, USA). A Chi-square independence test was performed to analyze the data. Additionally, to test the prevalence of a particular virulence factor among *E. faecalis* and *E. faecium* the Chi-square independence test with Yates correction or Fisher’s exact test for small samples (≤5) were used. P ≤ 0.05 was considered statistically significant.

## Supplementary information

Biofilm formation capacity and presence of virulence factors among commensal Enterococcus spp. from wild birds
