# Supplementary material for: Biofilm formation capacity and presence of virulence factors among commensal Enterococcus spp. from wild birds
Source: Sci Rep. 2019 Aug 1;9:11204. doi: 10.1038/s41598-019-47602-w (PMC6671946; doi:10.1038/s41598-019-47602-w)
Supplement: Supplementary file 1 — Biofilm formation capacity and presence of virulence factors among commensal Enterococcus spp. from wild birds [file 41598_2019_47602_MOESM1_ESM.pdf]

Supplementary Information

Biofilm formation capacity and presence of virulence factors among commensal *Enterococcus* spp. from wild birds

Dagmara Stępień-Pyśniak[1*], Tomasz Hauschild[2], Urszula Kosikowska[3], Marta Dec[1], Renata Urban-Chmiel[1]

[1]Department of Veterinary Prevention and Avian Diseases, Institute of Biological Bases of Animal Diseases**,** Faculty of Veterinary Medicine, University of Life Sciences in Lublin, Lublin, Poland. [2]Department of Microbiology, Institute of Biology, University of Bialystok, Białystok, Poland. [3]Department of Pharmaceutical Microbiology with Laboratory for Microbiological Diagnostics, Medical University in Lublin, Lublin, Poland. *Correspondence and requests for materials should be addressed to D.S.P. (email: dagmara.stepien@up.lublin.pl)

Origin[a]	Isolates	Strain number	Phenotype					Genotypic patterns of virulence factors
			Gelatinase	Beta-haemolysis	Biofilm in TSB +1% glucose[b]	Biofilm in BHI +2% glucose[b]	Hydrophobicity (%)	
White-tailed Eagle	*E. faecalis*	2B	+	-	II	IV	62.5	*ebpA, ebpB, ebpC, srt, gelE, sprE, efaAfs, fsrA, fsrB, fsrC, cpd, cob, ccf*
White-tailed Eagle	*E. faecalis*	3	+	-	IV	IV	100	*ebpA, ebpB, ebpC, pil,srt, ace, gelE, sprE, efaAfs, fsrA, fsrB, fsrC, cpd, cob, ccf*
Eurasian Tawny Owl	*E. faecalis*	8	+	-	II	IV	100	*ebpA, ebpB, ebpC, srt, ace, gelE, sprE, efaAfs, fsrA, fsrB, fsrC, cpd, cob, ccf*
Little Bittern	*E. faecalis*	11	+	-	II	IV	50	*ebpA, ebpB, ebpC, pil,srt, ace, gelE, sprE, efaAfs, fsrA, fsrB, fsrC, cpd, cob, ccf*
Hoopoe	*E. faecalis*	12	+	-	II	IV	70	*ebpA, ebpB, ebpC, pil,srt, ace, gelE, sprE, efaAfs, fsrA, fsrB, fsrC, cpd, cob, ccf*
Mallard	*E. faecalis*	20	+	-	I	I	0	*ebpA, ebpB, ebpC, pil,srt, ace, gelE, sprE, efaAfs, fsrA, fsrB, fsrC, cpd, cob, ccf*
Mallard	*E. faecalis*	25	+	-	II	IV	100	*ebpA, ebpB, ebpC, srt, ace, agg, asa1, gelE, sprE, efaAfs, fsrA, fsrB, fsrC, cpd, cob, ccf*
Mallard	*E. faecalis*	26	+	-	IV	I	0	*ebpA, ebpB, ebpC, srt, ace, gelE, sprE, efaAfs, fsrA, fsrB, fsrC, cpd, cob, ccf*
Common Blackbird	*E. faecalis*	28	+	-	III	I	0	*ebpA, ebpB, pil, srt, ace, gelE, sprE, efaAfs, fsrA, fsrB, fsrC, cpd, cob, ccf*
Eurasian Jay	*E. faecalis*	30	+	-	III	III	44.4	*ebpB, ebpC, ace, gelE, sprE, efaAfs, fsrA, fsrB, fsrC, cpd, cob, ccf*
Short-eared Owl	*E. faecalis*	31	+	-	I	III	100	*ebpA, ebpB, ebpC, srt, ace, gelE, sprE, efaAfs, fsrA, fsrB, fsrC, cpd, cob, ccf*

Eurasian Tawny Owl	*E. faecalis*	32	+	-	III	I	0	*ebpB, pil, srt, ace, gelE, sprE, efaAfs, fsrA, fsrB, fsrC, cpd, cob, ccf*
Mallard	*E. faecalis*	33	+	-	I	II	33.3	*ebpA, ebpB, pil,srt, ace, asa1, gelE, sprE, efaAfs, fsrA, fsrB, fsrC, cpd, cob, ccf*
Little Bittern	*E. faecalis*	35	+	-	I	III	50	*ebpA, ebpB, ebpC, srt, ace, gelE, sprE, efaAfs, fsrA, fsrB, fsrC, cpd, cob, ccf*
Eurasian Sparrow Hawk	*E. faecalis*	36	+	-	I	III	57.1	*ebpA, ebpB, ebpC, srt, ace, gelE, sprE, efaAfs, fsrA, fsrB, fsrC, cpd, cob, ccf*
Common Buzzard	*E. faecalis*	42	+	-	II	III	100	*ebpC, srt, ace, gelE, sprE, efaAfs, fsrA, fsrB, fsrC, cpd, cob, ccf*
Little Owl	*E. faecalis*	44	+	-	II	III	100	*ebpB, srt, ace, gelE, sprE, efaAfs, fsrA, fsrB, fsrC, cpd, cob, ccf*
White-tailed Eagle	*E. faecalis*	46	+	-	I	IV	55.6	*ebpA, ebpB, ebpC, srt, ace, agg, asa1, gelE, sprE, efaAfs, fsrA, fsrB, fsrC, cpd, cob, ccf*
Mallard	*E. faecalis*	48A	+	-	I	III	100	*ebpC, srt, ace, agg, asa1, gelE, sprE, efaAfs, fsrA, fsrB, fsrC, cpd, cob, ccf*
Mallard	*E. faecalis*	50	+	-	I	III	66.7	*srt, ace, agg, asa1, gelE, sprE, efaAfs, fsrA, fsrB, fsrC, cpd, cob, ccf*
Lesser Spotted Woodpecker	*E. faecalis*	53	+	-	III	III	50	*ebpA, ebpB, ebpC, srt, ace, agg, asa1, gelE, sprE, efaAfs, fsrA, fsrB, fsrC, cpd, cob, ccf*
European Green Woodpecker	*E. faecalis*	54	+	-	IV	III	42.9	*ebpA, ebpC, srt, ace, agg, asa1, gelE, sprE, efaAfs, fsrA, fsrB, fsrC, cpd, cob, ccf*
Eurasian Tawny Owl	*E. faecalis*	56	+	-	III	III	57.1	*ebpA, ebpB, ebpC, srt, ace, agg, asa1, gelE, sprE, efaAfs, fsrA, fsrB, fsrC, cpd, cob, ccf*
Great Spotted Woodpecker	*E. faecalis*	58	+	-	III	III	42.9	*ebpB, ebpC, srt, ace, agg, asa1, gelE, sprE, efaAfs, fsrA, fsrB, fsrC, cpd, cob, ccf*

Grey Heron	*E. faecalis*	61	+	-	II	II	100	*ebpA, ebpB, srt, ace, agg, asa1, gelE, sprE, efaAfs, fsrA, fsrB, fsrC, cpd, cob, ccf*
Western Marsh Harrier	*E. faecalis*	62	+	-	I	II	33.3	*ebpB, ebpC, srt, ace, gelE, sprE, efaAfs, fsrA, fsrB, fsrC, cpd, cob, ccf*
Grey Heron	*E. faecalis*	64A	+	-	II	III	100	*ebpA, ebpB, srt, ace, gelE, sprE, efaAfs, fsrA, fsrB, fsrC, cpd, cob, ccf*
Great Egret	*E. faecium*	1	-	-	I	I	14.3	*efaAfm, ccf*
White-tailed Eagle	*E. faecium*	2A	-	-	I	III	25	*efaAfm, ccf*
Mute Swan	*E. faecium*	5	-	-	I	III	10	*efaAfm, ccf*
Common Raven	*E. faecium*	10	-	-	I	III	37.5	*efaAfm, ccf*
Eurasian Golden Oriole	*E. faecium*	13	-	-	I	III	0	*efaAfm, ccf*
Eurasian Sparrow Hawk	*E. faecium*	14	-	-	I	IV	0	*efaAfm, ccf*
Common Buzzard	*E. faecium*	15	-	-	I	IV	44.4	*efaAfm, ccf*
Common Tern	*E. faecium*	16	-	-	I	IV	0	*ebpC, srt, ace, gelE, sprE, efaAfm, fsrA, fsrB, fsrC, cpd, ccf*
Mallard	*E. faecium*	17	-	-	II	IV	66.7	*efaAfm, ccf*
Mallard	*E. faecium*	19A	-	-	I	III	0	*efaAfm*
Mallard	*E. faecium*	19B	-	-	I	III	0	*efaAfm, ccf*
Mallard	*E. faecium*	22	-	-	I	III	0	*efaAfm, ccf*
Fieldfare	*E. faecium*	29	-	-	IV	II	0	*efaAfm, ccf*
Mallard	*E. faecium*	34	-	-	I	I	0	*ebpA, ebpC, srt, ace, gelE, sprE, efaAfm, fsrA, fsrB, fsrC, cpd, cob, ccf*
Mallard	*E. faecium*	38A	-	-	II	II	12.5	*ebpA, ebpC, srt, ace, gelE, sprE, efaAfm, fsrA, fsrB, fsrC, cpd, ccf*
Mallard	*E. faecium*	39A	+	-	II	III	0	*ebpA, srt, ace, gelE, sprE, efaAfm, fsrA, fsrB, fsrC, cpd, ccf*

Short-eared Owl	*E. faecium*	57	-	-	I	I	0	*ebpA, ebpC, srt, ace, agg, asa1, gelE, sprE, efaAfm, fsrA, fsrB, fsrC, cpd, cob, ccf*
European Green Woodpecker	*E. faecium*	60	-	-	I	II	10	*ebpC, srt, asa1, efaAfm, ccf*
White Stork	*E. hirae*	4	-	-	II	IV	22.2	*ebpC, ccf*
Mallard	*E. hirae*	23	-	-	I	III	0	-
Mallard	*E. hirae*	41	-	-	II	II	0	*srt, gelE, sprE, fsrA, fsrC, cpd, ccf*
Barn Owl	*E. hirae*	43	-	-	II	III	28.6	*ebpC, srt, asa1, gelE, sprE, fsrA, fsrC, cpd, cob, ccf*
Long-eared Owl	*E. hirae*	45	+	-	I	II	37.5	*ebpA, ebpC, pil, srt, ace, gelE, sprE, fsrA, fsrB, fsrC, cpd, cob, ccf*
Mallard	*E. durans*	24	-	-	I	III	22.2	*ccf*
Mallard	*E. durans*	37		-	I	II	42.9	*ccf*
Mallard	*E. casseliflavus*	49	-	-	I	IV	62.5	*ebpC, ccf*
Mallard	*E. casseliflavus*	52	-	-	I	III	42.9	*ebpC, ccf*

Table S1. Phenotypic and genotypic properties in *Enterococcus* strains isolated from wild birds.

[a] Western Marsh Harrier (*Circus aeruginosus*), White-tailed Eagle (*Haliaeetus albicilla*), Eurasian Sparrow Hawk (*Accipiter nisus*), Common Buzzard (*Buteo buteo*), Lesser Spotted Woodpecker (*Dendrocopos minor*), Great Spotted Woodpecker (*Dendrocopos major*), European Green Woodpecker (*Picus viridis*), Mute Swan (*Cygnus olor*), Mallard (*Anas platyrhynchos*), Barn Owl (*Tyto alba*), Little Owl (*Athene noctua*), Eurasian Tawny Owl (*Strix aluco*), Long-eared Owl (*Asio otus*), Short-eared Owl (*Asio flammeus*), Eurasian Golden Oriole (*Oriolus oriolus*), Little Bittern (*Ixobrychus minutus*), Grey Heron (*Ardea cinerea*), Great Egret (*Ardea alba*), Hoopoe (*Upupa epos*), Fieldfare (*Turdus pilaris*),

Common Blackbird (*Turdus merula*), Eurasian Jay (*Garrulus glandarius*), Common Raven (*Corvus coraz*), Common Tern (*Sterna hirundo*), White Stork (*Ciconia ciconia*)

[b] Category of biofilm formation: I- no biofilm; II- weak biofilm; III- moderate biofilm; IV- strong biofilm

....

Genes	Encoded virulence factor	Primer sequence 5' – 3'	Annealing temperature	Amplicon length (bp)	Reference
ebpA	endocarditis and biofilm-associated pili (fibre tip pilin)	F: CCATTTGCAGAAGCAAGAATG<br>R: GAGTGAAAGTTCCTCCTCTAG	54	613	1
ebpB	endocarditis and biofilm-associated pili (cell wall-anchored pilin)	F: CATTAGCAGAGGCATCGCAA<br>R: CAAGTGGTGGTAAGTCATAGG	54	504	1
ebpC	endocarditis and biofilm-associated pili (major shaft pilin)	F: CTGCTACGAATATGGTGGTG<br>R: GGTGTTTGATTGTTTGCTTC	54	487	1
pil	pili	F: GAAGAAACCAAAGCACCTAC<br>R: CTACCTAAGAAAAGAAACGCG	54	620	1
srt	pilus-associated sortase	F: GTATCCTTTTGTTAGCGATGC<br>R: TGTCCTCGAACTAATAACCGA	54	612	1
ace	collagen-binding protein	F: AAAGTAGAATTAGATCCACAC<br>R: TCTATCACATTCGGTTGCG	48	320	2
agg	aggregation substance	F: CACGTAATTCTTGCCCACCA<br>R: CAAGCATTATTGGCAGCGTT	55	520	3
asa1	aggregation substance	F: GCACGCTATTACGAACTATGA<br>R: TAAGAAAGAACATCACCACGA	56	375	4
esp	enterococcal surface protein	F: AGATTTCATCTTTGATTCTTGG<br>R: AATTGATTCTTTAGCATCTGG	56	510	4
$efaA_{fs}$	*E. faecalis* specific endocarditis antigen	F: GACAGACCCTCACGAATA<br>R: AGTTCATCATGCTGTAGTA	51	705	5

*efaA*fm	*E.faecium* specific surface antigen	F: AACAGATCCGCATGAATA<br>R: CATTTCATCATCTGATAGTA	48	735	5
gelE	gelatinase	F: ACCCCGTATCATTGGTTT<br>R: ACGCATTGCTTTTCCATC	48	419	6
sprE	serine protease	F: TTGAGCTCCGTTCCTGCCGAAAGTCATTC<br>R: TTGGTACCGATTGGGGAACCAGATTGACC	55	591	6
hyl	hyaluronidase	F: ACAGAAGAGCTGCAGGAAATG<br>R: GACTGACGTCCAAGTTTCCAA	56	276	4
fsrA	response regulator	F: ATGAGTGAACAAATGGCTATTTA<br>R: CTAAGTAAGAAATAGTGCCTTGA	49	740	6
fsrB	signalling peptide	F: GGGAGCTCTGGACAAAGTATTATCTAACCG<br>R: TTGGTACCCACACCATCACTGACTTTTGC	63	566	6
fsrC	histidine kinase	F: ATGATTTTGTCGTTATTAGCTACT<br>R: CATCGTTAACAACTTTTTTACTG	49	1343	6
cylA	cytolysin activator	F: ACTCGGGGATTGATAGGC<br>R: GCTGCTAAAGCTGCGCTT	56	688	4
cylB	transport of cytolysin	F: ATTCCTACCTATGTTCTGTTA<br>R: AATAAACTCTTCTTTTCCAAC	51	843	5
cylM	cytolysin synthetase	F: CTGATGGAAAGAAGATAGTAT<br>R: TGAGTTGGTCTGATTACATTT	53	742	5

*cylL*L	cytolysin precursor	F: GCTTCACCTCACTAAGTTTTATAG<br>R: GATGGAGGGTAAGAATTATGG	53	253	7
cpd	sex pheromone	F: TGGTGGGTTATTTTTCAATTC<br>R: TACGGCTCTGGCTTACTA	48	782	5
cob	sex pheromone	F: AACATTCAGCAAACAAAGC<br>R: TTGTCATAAAGAGTGGTCAT	51	1405	5
ccf	sex pheromone	F: GGGAATTGAGTAGTGAAGAAG<br>R: AGCCGCTAAAATCGGTAAAAT	52	543	5

Table S2. Target genes and primers used in this study.

1. Hashem, Y.A., Amin, H.M., Essam, T.M. & Aziz, R.K. Biofilm formation in enterococci: genotype-phenotype correlations and inhibition by vancomycin. *Sci Rep.* **7,** 5733 (2017).
2. Duprè, I., Zanetti, S., Schito, A.M., Fadda, G. & Sechi, L.A. Incidence of virulence determinants in clinical *Enterococcus faecium* and *Enterococcus faecalis* isolates collected in Sardinia (Italy). *J Med Microbiol.* **52,** 491-498 (2003).
3. Seputiene, V., Bogdaite, A., Ruzauskas, M. & Suziedeliene, E. Antibiotic resistance genes and virulence factors in *Enterococcus faecium* and *Enterococcus faecalis* from diseased farm animals: pigs, cattle and poultry. *Pol J Vet Sci.* 15, 431-438 (2012).
4. Vankerckhoven, V., et al. Development of a multiplex PCR for the detection of *asa1*, *gelE*, *cylA*, *esp*, and *hyl* genes in enterococci and survey for virulence determinants among European hospital isolates of *Enterococcus faecium*. *J Clin Microbiol.* **42,** 4473-4479 (2004).

5. Eaton, T.J. & Gasson, M.J. Molecular screening of *Enterococcus* virulence determinants and potential for genetic exchange between food and medical isolates. *Appl Environ Microbiol.* **67,** 1628-1635 (2001).

6. Lopes Mde, F., Simões, A.P., Tenreiro, R., Marques, J.J. & Crespo, M.T. Activity and expression of a virulence factor, gelatinase, in dairy enterococci. *Int J Food Microbiol*. **112,** 208-214 (2006).

7. Semedo, T., et al. Comparative study using type strains and clinical and food isolates to examine hemolytic activity and occurrence of the cyl operon in enterococci. *J Clin Microbiol*. **41,** 2569-76 (2003).

....
